# Nonlinear Superchiral Meta-Surfaces: Tuning Chirality and Disentangling Non-Reciprocity at the Nanoscale

**DOI:** 10.1002/adma.201401021

**Published:** 2014-04-17

**Authors:** V K Valev, J J Baumberg, B De Clercq, N Braz, X Zheng, E J Osley, S Vandendriessche, M Hojeij, C Blejean, J Mertens, C G Biris, V Volskiy, M Ameloot, Y Ekinci, G A E Vandenbosch, P A Warburton, V V Moshchalkov, N C Panoiu, T Verbiest

**Affiliations:** NanoPhotonics Centre, Cavendish Laboratory Department of Physics University of CambridgeJ. J. Thomson Avenue Cambridge CB3 0HE, UK E-mail: vkv23@cam.ac.uk; University Hasselt and transnational University LimburgBIOMED, Diepenbeek, Belgium; Electrical Engineering University College London Torrington PlaceLondon WC1E 7JE, UK; ESAT-TELEMIC, KU LeuvenB-3001, Leuven, Belgium; Electrical Engineering University College London Torrington PlaceLondon WC1E 7JEUK London Centre for Nanotechnology University College London17–19 Gordon St, London WC1H 0AH, UK; Molecular Electronics and PhotonicsKU Leuven BE-3001, Belgium; Laboratory for Micro and Nanotechnology Paul Scherrer Institute5232, Villigen-PS, Switzerland; Department of Physics West University of Timisoara B-dul Vasile ParvanNr. 4, Timisoara, 300223, Timis, Romania; Nanoscale Superconductivity and Magnetism & Pulsed Fields Group INPAC, KU LeuvenCelestijnenlaan 200 D B-3001, Leuven, Belgium; Electrical Engineering, University College London Torrington PlaceLondon WC1E 7JE, UKThomas Young Centre London Centre for Nanotechnology University College London17–19 Gordon St, London, WC1H 0AH, UK

**Keywords:** chirality, surface plasmon resonance, plasmonics, metamaterials, second harmonic generation

Due to the favorable power-law scaling of near-field enhancements, the nonlinear optical properties of chiral plasmonic nano- and metamaterials are of prime fundamental and practical interest. However, these optical properties remain largely unexplored. Here we demonstrate that nonlinear chiroptical effects are sensitive to superchiral light enhancements and can therefore be used to guide the design of superchiral devices for enhanced chiroptical sensing and asymmetric molecular synthesis or catalysis. While maximal response in linear chiral metamaterials is achieved for deep sub-wavelength dimensions, we show that the chiral coupling in the nonlinear case has a local maximum for a distance of half the second harmonic wavelength. Fundamentally, whereas conservation under space and time reversal causes chiral linear metamaterials to be reciprocal, we demonstrate that the nonlinear ones are non-reciprocal. These results provide a framework for exploiting the benefits of chiral nonlinear meta-surfaces.

Chirality represents the handedness of Nature, the property where the mirror image of an object cannot be superimposed on the object itself. Although often associated with biochemistry due to the numerous chiral bio-molecules, recently chirality has attracted much attention in relation to metamaterials, which consist of metallic nanostructures with substantially sub-wavelength features. Chiral metamaterials appear very promising because of their large optical activity, potential for slow light applications, negative refractive index,[1,2] and broadband circular polarizers,[3] as well as repulsive Casimir forces that can lead to nano-levitation and frictionless nano-motors.[4] Additionally, chiral metamaterials can compress the helical pitch of circularly polarized light thereby achieving superchiral light.[5] Just as plasmonic local field enhancements can be used to increase the interaction of light with the electronic, vibrational and rotational resonances of molecules (up to 14 orders of magnitude[6,7] in the case of Raman scattering),[8] superchiral light can increase the interaction with *chiroptical* (chiral optical) resonances. Recently, various aspects of superchiral light have been theoretically investigated,[9,10] and the chiroptical interactions between molecules and plasmonic nanostructures are becoming the subject of growing interest.[11–15] Chiral metamaterials could therefore be used to increase the enantioselectivity at surfaces,[5] as well as to achieve asymmetric synthesis in chiral photochemistry. Because their optical response is proportional to multiple powers of the enhanced optical near-field intensity, chiral metal nanostructures also constitute excellent candidates for nonlinear optical materials with new or improved properties.[16,17]

In optical second harmonic generation (SHG) two photons at a fundamental frequency (FF) are annihilated to generate a single photon at twice the frequency (half the wavelength).[18] Surface SHG can be interface-sensitive down to the atomic monolayer, its signal being greatly enhanced by the presence of surface plasmons,[19] i.e. the coherent oscillations of free electrons confined at metal/dielectric interfaces.[20] Moreover, the chiroptical effects in SHG are typically three orders of magnitude larger than their linear optical counterparts.[21,22] Nonlinear metamaterials and meta-surfaces are therefore expected to achieve record-high chiroptical values compared to those of natural materials and consequently to serve as highly sensitive probes for exploring chiral molecular chemistry. Presently though, very little has been done towards fulfilling these high expectations. Basic understanding of the nonlinear chiroptical properties of meta-surfaces is also lacking; for instance, fundamental physical properties, such as reciprocity, have not yet been investigated. The question of reciprocity in meta-surfaces is crucial as they are the basic building blocks of bulk metamaterials.

For our studies, we chose traditional Slavic symbols, which provide an abundance of chiral designs.[23] Upon reproducing such designs at the nanoscale, we reveal for the first time a relation between SHG and superchiral light. Superchiral light has previously only been investigated theoretically or by its effect on chiral molecules. Our data suggest that SHG provides the means for experimentally mapping superchiral light. This technique can thus be employed to guide the design of plasmonic superchiral devices, with the ability to probe the chirality of molecules analogous to the way plasmonic surface-enhanced Raman scattering (SERS) substrates can probe the Raman spectra of molecules. We optimize the nonlinear meta-surface by tuning the gap size between individual nanostructures within a unit cell for maximal nonlinear response. Finally, at the fundamental level, we show that whereas chiroptical effects from meta-surfaces in the linear optical regime are reciprocal, in the nonlinear regime these effects can be non-reciprocal. Counter-intuitively, this non-reciprocal behavior is nevertheless consistent with three-dimensional chirality.

We begin our investigation with designs inspired from the traditional Slavic symbol 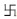, called fash (фaш)[24] and its mirror-image 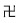, called *agni* (агни). The latter is composed of 4 L-shaped nanostructures, made of gold, see pink highlight in **Figure** **1**a. Each nanostructure is 60 nm thick, 1 μm long and the strip is 200 nm wide. The gap between the nanostructures at the chiral center of the unit cell form a square of side 200 nm. First, we present the SHG response from these nanostructures and then demonstrate its relation to superchiral light.

**Figure 1:**
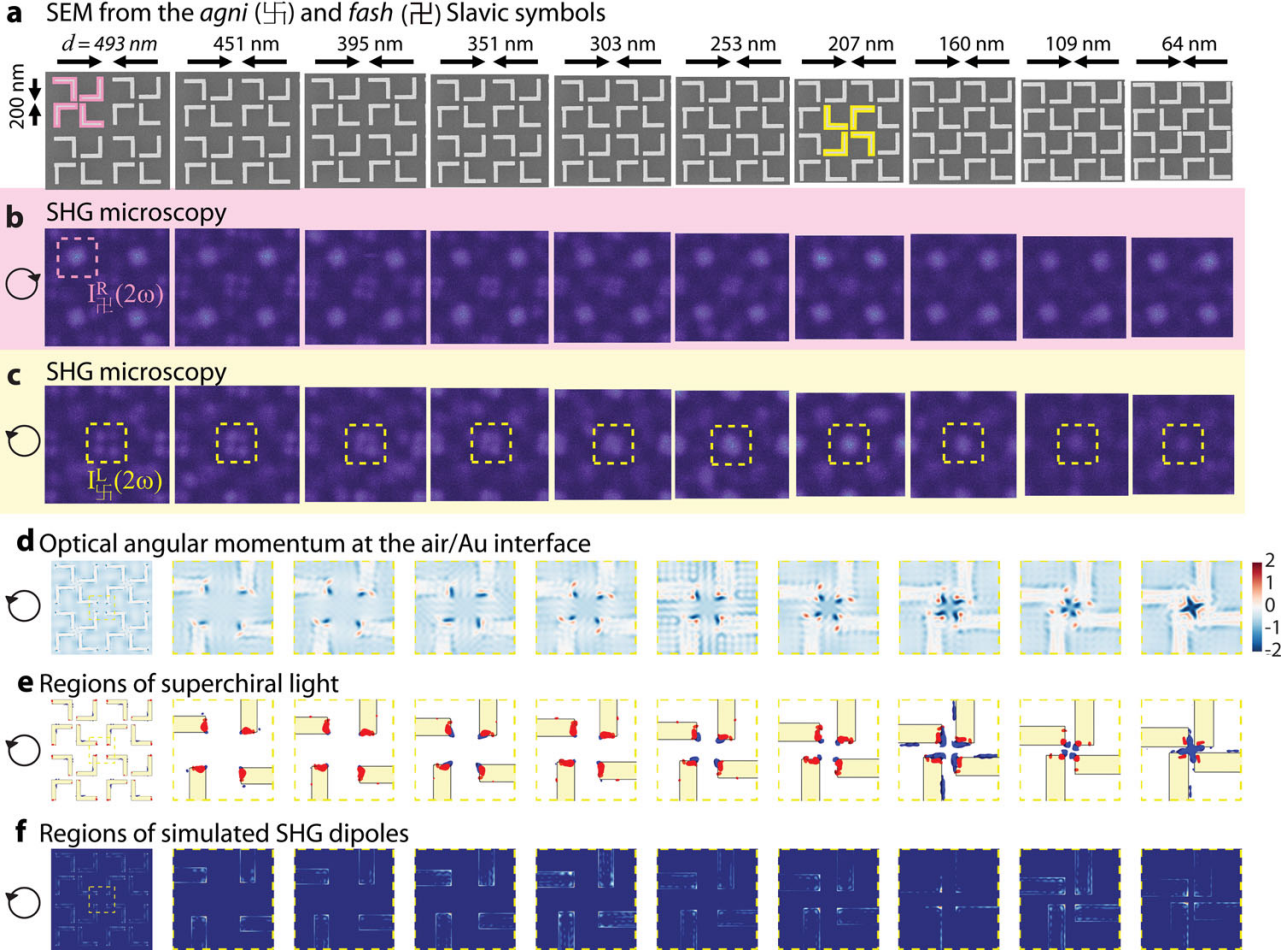
Simulations show that chiroptical SHG signals are associated with the enhanced optical chirality. (a), SEM images of gold nanostructures forming 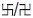 unit cells. The arrows indicate the varying gap between unit cells *d*. (b)-(c), SHG microscopy of the nanostructured arrays, for *L* and *R* illumination at the wavelength of 900 nm, respectively. The images show strong SHG emission from the chiral centers of the 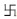 and 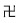 meta-molecules. (d), Simulated distribution of optical angular momentum (OAM) at the air/Au interfaces, normalized to that of the incoming light. Strong OAM density is observed at *d* in the range 160–253 nm, giving rise to localized superchiral light enhancements, in (e). The red and blue regions represent local fields of opposite handedness, where C > 1 is left-handed and C < -1 is right-handed. (f), Simulated spatial distribution of the nonlinear polarization which gives rise to SHG. The SHG clearly matches the regions where the density of OAM is enhanced.

The plasmonic meta-surface consists of two interspersed lattices of chiral meta-molecules with opposite chirality. The incoming light at the FF induces in each meta-molecule a nonlinear SHG dipole, which generates the measured signal. The amplitude of the nonlinear dipoles excited by optical frequency *ω* is given by 
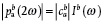
, where *a* = 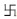 or 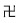 corresponds to the chiral centers (meta-molecules), *b* =*L* or *R* corresponds to the direction of circularly polarized light and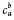 are coupling coefficients. In Figure 1, the chiral centers 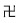 and 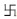 are marked in pink and yellow, respectively. The intensity of the SHG seen in Figures 1(b,c) is thus 
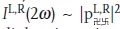
. The difference in SHG intensity for *L* and *R* light gives rise to an SHG – circular dichroism (SHG-CD) effect. The strength of the SHG-CD effect is quantified by the ratio:


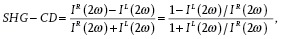
(1)

where *I^L^* and *I^R^* indicate the intensity for *L* and *R* light, respectively. Note that in our experiments 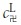 and 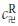 are constant (fixed separation) whereas 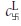 and 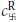 vary with the unit cell separation distance *d*. It should also be noted that the chiral coupling between the individual nanostructures occurs both diagonally and side-to-side, as is illustrated by Figure S1 (Supplementary Information). Moreover, the coupling is very robust versus sample thickness, wavelength and geometry (Figure S2). These experiments also demonstrate that the data in Figure 1 correspond indeed to SHG and not to two-photon luminescence, seen from emission spectra for arrays with 200 nm separation between the nanostructures (Figure S3). For reference, the distribution of optical near-fields at the FF on the surface of the nanostructures can be seen in Figure S4. Next, we establish the relation between the spatial distribution of SHG and that of superchiral light at the FF.

On the one hand, the chirality of the electromagnetic field is defined as: 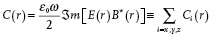,[25] where 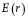 and 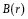 are the electric and magnetic fields (at the FF), respectively, 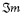 means the imaginary part, and 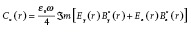; 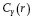 and 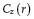 are obtained from this relation by index permutation. These quantities are related to the spin part of the angular momentum flux density:[26]


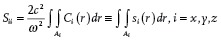
(2)

where 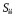 is the spin part of the *i*th component of the total angular momentum flux through a plane whose normal is oriented along the *i*th axis and 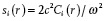 is the corresponding flux density. Figure 1d shows the simulated optical angular momentum flux density 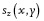, at the air/Au interface, while in Figure 1e we map the regions with superchiral field, 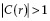, i.e. regions where the chirality is larger than that of circularly polarized light. The coordinate system is oriented so that *x* and *y* are in the plane of the sample, with *y* along the direction of S-polarization, while *z* is perpendicular to the sample. The plots in Figure 1e and Figure S5 correspond to *L* and *R* incident light, respectively.

On the other hand, the polarization at the second harmonic frequency can be written as:


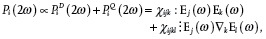
(3)

where 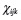 and 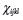 are third- and fourth-rank susceptibility tensors, giving rise to dipolar 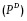 and quadrupolar 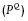 SHG polarizations, respectively. The indices *i*, *j, k* and *l* represent any of the Cartesian coordinates *x*, *y,* and *z*. Note that the fields in Equation 3 are local fields and not the incident ones.

For chiral light, the dissymmetry factor, *g*, measures the fractional difference in rates of molecular excitation between *L* and *R* light. If only the electric dipole-magnetic dipole interaction is considered, the dissymmetry factor is given by *g* = *cC*/2*U_e_ω*,[5] where *U_e_* is the local electric energy density. The electric dipole-electric quadrupole interaction has an additional contribution to the dissymmetry factor, proportional to *q_ijk_* = *E_i_*∇*_j_E_k_*,[5] which is identical to the quadrupolar contribution to the SHG. Hence, it is indeed expected that superchiral light causes an enhanced contribution to the SHG-CD coming from the quadrupolar term in Equation 3. Due to the strong enhancement of the local optical fields, plasmonic nanostructures are ideal systems to achieve both superchiral light and enhancement of quadrupolar contributions to SHG, and consequently provide an effective test bed to study SHG-CD effects. Equally important, it follows that SHG can constitute a valuable tool for probing the regions of superchiral light enhancements on plasmonic meta-surfaces. The dipolar term in Equation 3 is very sensitive to near-field enhancements, as experimentally observed,[19,27] and here explicitly demonstrated by the spatial distribution of the nonlinear polarization, determined from first principles calculations (Figure 1f and discussion in the Supplementary Information). Specific polarizer-analyzer configurations can be used to separate the dipolar from the quadrupolar contributions to the detected SHG signal.

Having elucidated the relation between the nonlinear chiroptical effect and superchiral light, we use this knowledge to design a meta-surface that is particularly suited to study nonlinear chiroptical phenomena. First, we determine the separation distance for optimal nonlinear response and then we provide a more effective design for achieving a large macroscopic SHG-CD.

Within the yellow highlighted region in Figure 1c, the non-monotonic variation of SHG intensity as a function of the gap distance is not surprising as *c^b^_a_* are negligible when *d* is very large or very small. Indeed considering the extreme cases, for very small gaps SHG contributions from opposite interfaces interfere destructively,[28,29] whereas for very large gaps, there is no longer chiral coupling. A non-monotonic intensity dependence of SHG with respect to gap sizes has already been reported in the case of achiral split ring metamaterials.[30] In contrast, here we achieve a non-monotonic SHG-CD effect, as a function of the gap size. We tune the SHG-CD by simply tuning *d* and ensuring that the circularly polarized light does not couple with the same efficiency to the two types of chiral meta-molecules (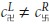). It should be noted that the SHG-CD maximum is situated near *d* = 225 nm, which corresponds to half the SHG wavelength. An intuitively appealing explanation for this maximum can be found upon considering the orientation of the air/Au interfaces in neighboring nanostructures. These interfaces, facing each other, are identical but have opposite orientations. Consequently, SHG light scattering from one interface and travelling over a distance of half the wavelength interferes constructively with SHG light from the opposite interface.

In **Figure** **2**a, the ratio 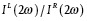 is plotted versus the separation distance *d*. Each experimental point on the graph is the result of an intensity average per pixel over 9 chiral centers. A pronounced SHG-CD effect can be observed as the yellow and pink points are clearly separated by the 
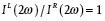
 line. An alternative way of displaying the SHG-CD peak is by examining the SHG intensity for each unit cell under illumination from the matching circularly polarized light. The ratio 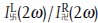) should then be equal to 1 when both unit cells have identical dimensions, as in this case 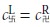. In Figure 2b, it can be seen that this is indeed true. The plot indicates that the maximum chiroptical effect is situated at *d*∼200 nm.

**Figure 2:**
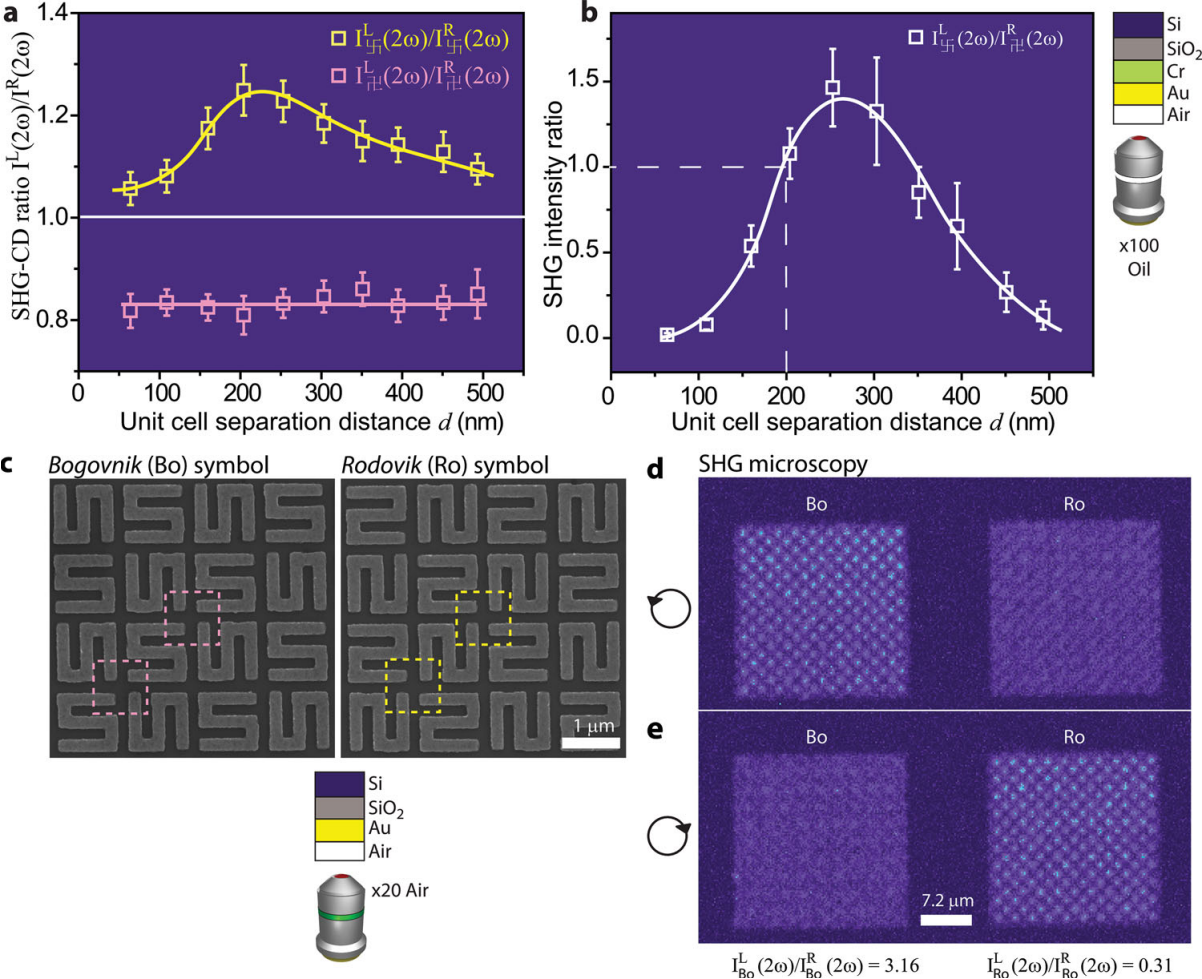
Optimizing the nonlinear chiroptical response of the meta-surface by gap size and design. (a), SHG-circular dichroism (SHG-CD) from the and chiral centers displayed as a function of the gap size *d*. (b), Ratio of SHG intensity 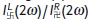 as a function of the gap size *d*. (c), SEM images of the Bogovnik (Bo) and Rodovik (Ro) traditional Slavic symbols. Contrary to the 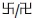 geometry, the Bo/Ro geometry consists of identical (and not mirrored) interspaced unit cells. (d)-(e), SHG microscopy of the Bo and Ro sample arrays, upon illumination with left- and right-hand circularly polarized light, respectively.

Whereas the interspaced 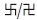 geometry clearly cannot be employed for large-area characterization, since the contributions from different unit cells cancel out, interspersing composite unit cells can also be employed to reinforce the chiral geometry of the meta-surface. This is illustrated by the nanostructures in Figure 2c, which are inspired by the S-like Slavic symbols *Bogovnik* (Боговник) and *Rodovik* (Родовик). The effects of this reinforcement are visible in the SHG microscopy images of the nanostructures, illuminated at 900 nm with *L* and *R* light (Figure 2d,e respectively). There is sharp contrast in the SHG emission from the sample arrays in Figure 2d, with the contrast reversing in Figure 2e. The SHG-CD from these meta-surfaces is over 52%, which is the largest value that has been reported to date. It is significantly above the 15% from twisted crosses[31] or the 10% from G-shaped nanostructures,[32] and it exceeds the 40% observed in star-shaped nanostructures.[33] It should be pointed out that in all the previous studies, the sample arrays did not constitute a continuous meta-surface but rather a collection of separated (uncoupled) nanostructures or unit cells. Because of this large chiroptical response, the meta-surface geometry presented in Figure 2c is also very convenient for macroscopic characterization.

The question of optical reciprocity is summarized in **Figure** **3**a and its dependence on space and time reversal transformations is presented in Figure S6 of the Supporting Information. Upon writing a chiral symbol, such as Ro in ink on a sheet of paper and then turning the page to look at the symbol from the opposite side, we recognize the opposite handedness of the symbol, i.e. Bo. Light however senses things differently as the symbol is not truly 2D – the symmetry in the third direction is broken due to the presence of paper on one side of the ink and air on the other. To demonstrate this at the nanoscale, we prepared Ro/Bo meta-surfaces by EBL, on a glass substrate. The arrays were each fabricated as two squares of side 3 mm. The reflection and transmission spectra (Figure S7a) and the numerical simulations (Figure S7b) reveal a broad resonance around 800 nm. At 800 nm, there is also a large linear chiroptical response, see CD-spectra in Figure 3b. The CD-spectra were acquired both for light incident from the air/Au and from the glass/Au interfaces. These spectra indicate linear CD values that are typical for chiral plasmonic nanostructures. Moreover, they demonstrate that the linear chiroptical response is reciprocal, i.e. upon flipping the sample, the chiroptical response does not change sign. This behavior is well known in the linear regime.[34] More specifically, the linear chiroptical response is reciprocal for the beam transmitted in the zero-order diffraction mode. Non-reciprocal behavior has actually been reported in higher-order diffracted modes[35] (this non-reciprocal behavior is also observed in diffraction from our samples, see Figure S8). However, the reciprocity of chiroptical behavior in meta-surfaces has never been investigated in the nonlinear regime.

**Figure 3:**
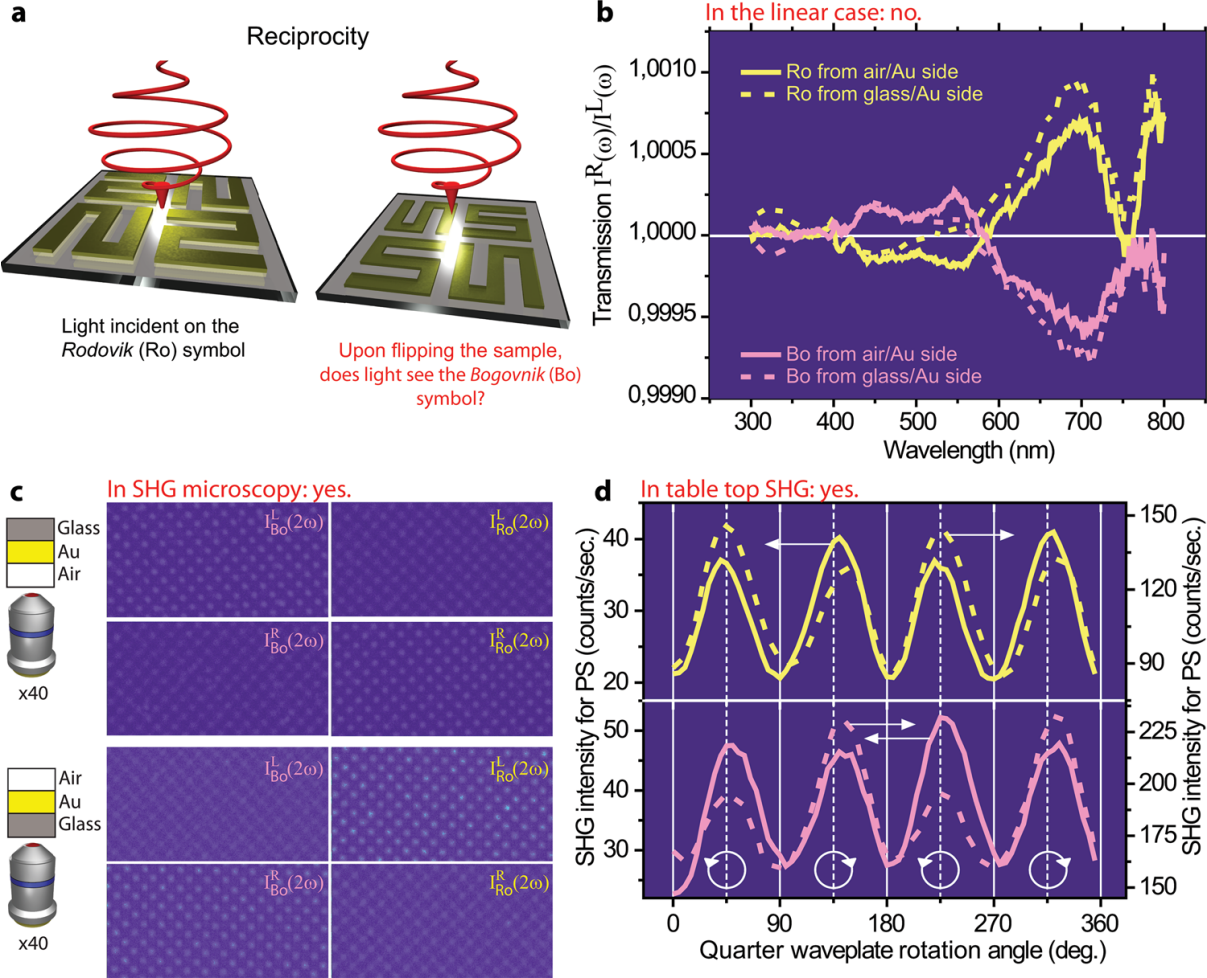
The meta-surface exhibits a reciprocal linear and a non-reciprocal non-linear chiroptical behavior. (a), Schematic diagram of reciprocity. (b), Linear circular dichroism (CD) spectra show the amplitude of the CD effect and demonstrate that the CD does not change sign upon flipping the sample (reciprocal chiroptical behavior). In the nonlinear regime however the SHG-CD does change sign. (c), Microscopic SHG images of the Bo and Ro geometries, acquired for light incident from the air/Au (upper panels) and from the glass/Au (lower panels) interfaces. (d), Macroscopic SHG intensity as a function of quarter waveplate rotation angle, with light incident from the air/Au (solid lines) and the glass/Au (dashed lines) interfaces. The polarizer-analyzer configuration was P-S. Both the microscopic and the macroscopic SHG-CD are non-reciprocal.

Figure 3c shows SHG images of the Ro- and Bo-shaped meta-surfaces, upon illumination with *L* and *R* light, through both the air/Au and the glass/Au interfaces. The images in Figure 3c demonstrate a clear contrast in the SHG emission, depending on the direction of circularly polarized light and on the handedness of the sample. Moreover, this contrast reverses upon flipping the meta-surface. Therefore, unlike the linear case, the SHG-CD response from chiral meta-surfaces is non-reciprocal. In order to eliminate any contributions from higher-order diffraction beams to our SHG-CD signal, the samples were macroscopically characterized in a table-top experimental configuration where only the transmitted beam in the zero-order diffraction was allowed to reach the detector.

Figure 3d presents the macroscopic SHG intensity as a function of the rotation angle of a quarter-waveplate. The waveplate angles for obtaining *L* and *R* light are indicated with oriented circles. The Ro/Bo arrays were investigated with light incident from both the air/Au and the glass/Au interfaces. For these measurements, the influence of rotational anisotropy on the results was eliminated by rotational averaging to specifically address chirality. Each curve presented in Figure 3d is the averaged response of 72 individual SHG intensity measurements versus waveplate rotation. The 72 iterations were acquired upon azimuthally rotating the sample over 360° in steps of 5°. This procedure allows us to reduce the symmetry of the meta-surface to isotropic chiral. As a result, in the P_IN_-S_OUT_ polarizer-analyzer configuration, and in the dipole approximation, the chirality is described by a single tensor component: 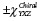. The value of this “chiral” tensor component changes sign depending on the handedness of the meta-surface and depending on the direction of circularly polarized light. In the case of P_IN_-S_OUT_, upon rotating a quarter waveplate, the SHG signal originates from the interference of two contributions, described by one “chiral” and one “achiral” (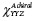) tensor component, i.e.[36]


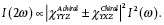
(4)

It is clear therefore that a contrast in the SHG signal (and SHG-CD) arises from reversing the handedness of the meta-surface or that of incident light. In Figure 3d, the SHG-CD effect is unambiguous upon switching the direction of circularly polarized light for both the Ro and Bo meta-surfaces. The SHG-CD effect also reverses depending on whether light is incident from the air/Au or from the glass/Au interface. It follows that this macroscopic investigation of the meta-surfaces confirms their non-reciprocal nonlinear chiroptical behavior. It could be argued that because the SHG signal originates from a meta-surface, it has the characteristics of an actual 2D response and hence the non-reciprocity that we observed. However, as discussed below, we argue that this is not the physical mechanism at work.

Although the highly interface-specific SHG might lead us to believe that the technique probes 2D chirality, the SHG signal originates from 3D *oriented* interfaces. Flipping the sample can be expressed as two possible symmetry transformations, either (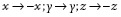) or (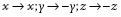). Under both transformations, the “chiral” tensor component does not change sign, which is consistent with the 3D chiral nature of the sample. The “achiral” component however does change sign under both transformations. It follows that whereas the SHG-CD effect is described by Equation 4, the non-reciprocal behavior arises from:


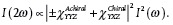
(5)

Since Equations 4 and 5 are mathematically identical, it follows that, for SHG, flipping a chiral sample is equivalent to reversing the handedness of the material or that of the incident light. Moreover, this statement is general as our analysis is valid for all tensor components of the isotropic chiral meta-surface.[37]

It should be emphasized that in all our results, the SHG is a *surface effect* intrinsically arising from chirality at a scale well below the necessity for phase-matching. This is very different from previous work on reciprocity and optical diodes,[38,39] which involves propagation effects, where phase-matching conditions are essential.

Furthermore, it is interesting to consider the possible effects of the meta-surfaces in Figure 1 on the chiroptical response of molecules. For a large number of randomly oriented chiral molecules deposited within the chiral center of the meta-molecules, we can expect the linear molecular response to increase by 100%, compared to that for circularly polarized light, regardless of whether the molecules are mobile within a liquid or immobilized on the meta-surface. This estimation is based on the contributions from electric dipoles only and could be enlarged upon including higher order multipoles. However, it should be pointed out that our meta-surfaces are not optimized for their linear optical properties. Although more difficult to foresee, the nonlinear chiroptical enhancement should be quite dramatic, since the nonlinear chiroptical response of molecules is typically three orders of magnitude larger than that of the linear chiroptical response.

In summary, we have presented a thorough investigation of the properties of nonlinear chiral meta-surfaces. We establish for the first time the relation between superchiral light and SHG, we design a nonlinear meta-surface with large chiroptical response, and we demonstrate that nonlinear meta-surfaces are fundamentally non-reciprocal. Because SHG originates in the near-field, it can guide the design of nanostructured plasmonic devices for increasing the chiroptical response of molecules. The diffraction limit could be circumvented through implementing a SNOM geometry.[40] Three applications based on this work can be readily envisioned. First is chiral sensing: based on the enhanced chiroptical interaction between molecules and chiral plasmonic centers, molecular enantiomer sensing could be significantly improved. Second is chiral separation: following a chemical synthesis, high intensity laser light could be employed to selectively destroy the undesired enantiomer. Third is direct asymmetric photosynthesis: with the increase in chiroptical interaction the yields of such sythesis[41] could be significantly enlarged.

## Experimental Section

*Sample Preparation*: Samples were fabricated on 170 μm thick glass substrates, cleanroom cleaned and treated with oxygen plasma. The substrates were coated with a 100 nm thick layer of 600 K poly(methyl methacrylat) (PMMA) and then baked at 180 °C for 5 min. A 30 nm Cu layer was evaporated on the top of PMMA as a conductive layer for the Electron Beam Lithography (EBL) exposure. The samples with the designed structure were exposed with an EBL tool (Vistec EBPG5000+) at 100 keV electron energy. Following the exposure, the Cu layer was removed in a nitric acid solution and the samples were then developed in a solution made of 1:3 Methyl isobutyl ketone (MIBK) and isopropyl alcohol (IPA) for 45 s. Finally, the fabrication was completed by evaporation of Cr/Gold, 3 nm/40 nm layer on the top of samples and a slow lift-off process in acetone was done in order to remove the PMMA and followed by an ultrasonic bath in order to remove all the remaining PMMA. The samples were then rinsed by IPA and dried in a flow of nitrogen gas. For the samples prepared on Si substrate, the preparation method is essentially the same, with Au thickness as specified in the text.

*SHG Experiments*: SHG microscopy images are collected with a confocal laser scanning microscope, Zeiss LSM 510 META (Jena, Germany). The sample is illuminated by a femtosecond pulsed Ti:Sapphire laser, directed to the sample by a dichroic mirror (HFT KP650) and through a Zeiss 20x Plan-APOCHROMAT or a 40x LD ACHROPLAN air objectives. The fundamental excitation wavelength is 900 nm. The working distance of the objectives extends to 0.6 mm and 1.8 mm, for the x20 and x40 objective and the laser spot on the sample is 670 nm and 580 nm half-width at 1/e^2^, respectively. After passing through a dichroic mirror (NFT545) and a short-pass filter (KP685), the SHG-signal is collected by a photomultiplier tube. The image is formed with a scanning speed of 102.4 μs for the pixel dwell time; each line is scanned twice and averaged.

The SHG table-top experiments are performed at 800 nm, with a Mai-Tai femtosecond laser system. A Glan-Laser polarizer insures the linear polarization of the incoming beam, before it passes a quarter waveplate that is mounted on an automatic rotation stage. The fundamental light, having a power of 80 mW, is then focused on the sample with an achromatic doublet lens, with focal distance of 10 cm, to a spot approximately 40 microns in diameter. The sample was oriented normally to the beam. The forward generated 400 nm photons are subsequently filtered through a BG39 filter that blocks the fundamental beam and are detected with a cooled photomultiplier tube. Finally, after pre-amplification, the intensity of the 400 nm signal is evaluated with a photon counter. For the purpose of our experiments, the sample is mounted on a motorized rotation stage, which enables the measurement of SHG (photons/sec.) at different rotation angles.

*Numerical Simulations*: MAGMAS is a numerical software tool, initially developed at the KU Leuven, for electromagnetic problems in the microwave and millimeter wave frequency bands.[42,43] Subsequently, it has been extended to include the special features of plasmonic nano-technology: (near) optical frequencies, strongly dispersive materials and volumetric meshing. It uses the method of moments for solving integral equations for the nanostructures. MAGMAS is a quasi-3D solver, which means that it is especially suited for topologies involving nano-components in multilayered structures. Compared to differential equation techniques, like for example FDTD, the resulting size of the numerical problem may be orders of magnitude smaller.

The field distribution at the fundamental frequency is calculated by using a commercially available software tool, RSoft's DiffractMOD.[44] The code implements a numerical method widely used in the analysis of optical properties of diffraction gratings, namely the rigorous coupled-wave analysis method. It consists in decomposing in Fourier modes the reflected and transmitted fields as well as the distribution of the dielectric constant, the corresponding modal amplitudes being determined by integrating across the structure a system of ordinary differential equations defined by the boundary conditions at the input and output facets of the periodic structure. The Fourier modes consist of both propagating and evanescent waves, which allows one to rigorously determine the spatial profile of the near-field and the energy content of the diffracted far-field. In our simulations, numerical convergence has been reached if 26 Fourier modes (diffraction orders) were used for each transverse dimension. Moreover, we assumed that the dielectric constant of gold is described by the Lorentz-Drude model, with the interband effects being described by a superposition of four Lorentzians.[45]

*Linear Optical Characterization*: The UV-Vis measurements were carried out with a Perkin Elmer Lambda 900 UV/VIS/NIR Spectrometer. The CD-spectra were acquired with a Jasco J-810 Spectropolarimeter. The goniometer data are obtained with a home-built rig featuring an Ocean Optics Spectrometer (QE65000) with detection range: 200–980 nm, the light source was an incandescent white light lamp with spectral range 200–2000 nm. The limiting factor for the wavelength range of the equipment was the wideband achromatic quarter waveplate 500–900 nm (B. Halle). Light was focused on the sample to a spot of approximately 1 mm in diameter.

## Supporting Information

As a service to our authors and readers, this journal provides supporting information supplied by the authors. Such materials are peer reviewed and may be re-organized for online delivery, but are not copy-edited or typeset. Technical support issues arising from supporting information (other than missing files) should be addressed to the authors.

Supplementary information accompanies this manuscript. Supporting Information is available from the Wiley Online Library or from the author.
