# Supplementary material for: Nonlinear Superchiral Meta-Surfaces: Tuning Chirality and Disentangling Non-Reciprocity at the Nanoscale
Source: Adv Mater. 2014 Apr 17;26(24):4074–81. doi: 10.1002/adma.201401021 (PMC4173128; doi:10.1002/adma.201401021)
Supplement: Supplementary file 1 — Supplementary [file adma0026-4074-SD1.pdf]

Copyright WILEY-VCH Verlag GmbH & Co. KGaA, 69469 Weinheim, Germany, 2014.

ADVANCED MATERIALS

Supporting Information

for *Adv. Mater.*, DOI: 10.1002/adma.201401021

Nonlinear Superchiral Meta-Surfaces: Tuning Chirality and Disentangling Non-Reciprocity at the Nanoscale

V. K. Valev, J. J. Baumberg, B. De Clercq, N. Braz, X. Zheng, E. J. Osley, S. Vandendriessche, M. Hojeij, C. Blejean, J. Mertens, C. G. Biris, V. Volskiy, M. Ameloot, Y. Ekinci, G. A. E. Vandenbosch, P. A. Warburton, V. V. Moshchalkov, N. C. Panoiu, and T. Verbiest*

SUPPLEMENTARY INFORMATION

Nonlinear superchiral meta-surfaces: tuning chirality and disentangling non-reciprocity at the nanoscale

By V. K. Valev, J. J. Baumberg, B. De Clercq, N. Braz, X. Zheng, E. J. Osley, S. Vandendriessche, M. Hojeij, C. Blejean, J. Mertens, C.G. Biris, V. Volskiy, M. Ameloot, Y. Ekinci, G. A. E. Vandenbosch, P. A. Warburton, V. V. Moshchalkov, N. C. Panoiu, T. Verbiest*

[*] Dr. V. K. Valev, C. Blejean, J. Mertens, Prof. J. J. Baumberg,
Cavendish Laboratory, Department of Physics, University of Cambridge, J. J. Thomson Avenue, Cambridge CB3 0HE, UK
E-mail: (vkv23@cam.ac.uk)
B. De Clercq, Prof. M Ameloot
University Hasselt and transnational University Limburg, BIOMED, Diepenbeek, Belgium
N. Braz,
Electrical Engineering, University College London, Torrington Place, London WC1E 7JE, UK
X. Zheng, Dr. V. Volskiy, Prof. G.A.E. Vandenbosch
ESAT-TELEMIC, KU Leuven, B-3001 Leuven, Belgium
Dr. E. J. Osley, Prof. P. A. Warburton
Electrical Engineering, University College London, Torrington Place, London WC1E 7JE, UK
London Centre for Nanotechnology, University College London, 17-19 Gordon St, London, WC1H 0AH, UK
S. Vandendriessche, Prof. T. Verbiest
Molecular Electronics and Photonics, KU Leuven, BE-3001, Belgium
Dr. M. Hojeij, Dr. Y. Ekinci
Laboratory for Micro and Nanotechnology, Paul Scherrer Institute, 5232 Villigen-PS, Switzerland
C. G. Biris
Department of Physics, West University of Timisoara, B-dul Vasile Parvan, Nr. 4, Timisoara 300223, Timis, Romania
Prof. V. V. Moshchalkov
Nanoscale Superconductivity and Magnetism & Pulsed Fields Group, INPAC, KU Leuven, Celestijnenlaan 200 D, B-3001 Leuven, Belgium
Dr. N. C. Panoiu
Electrical Engineering, University College London, Torrington Place, London WC1E 7JE, UK
Thomas Young Centre, London Centre for Nanotechnology, University College London, 17-19 Gordon St, London, WC1H 0AH, UK

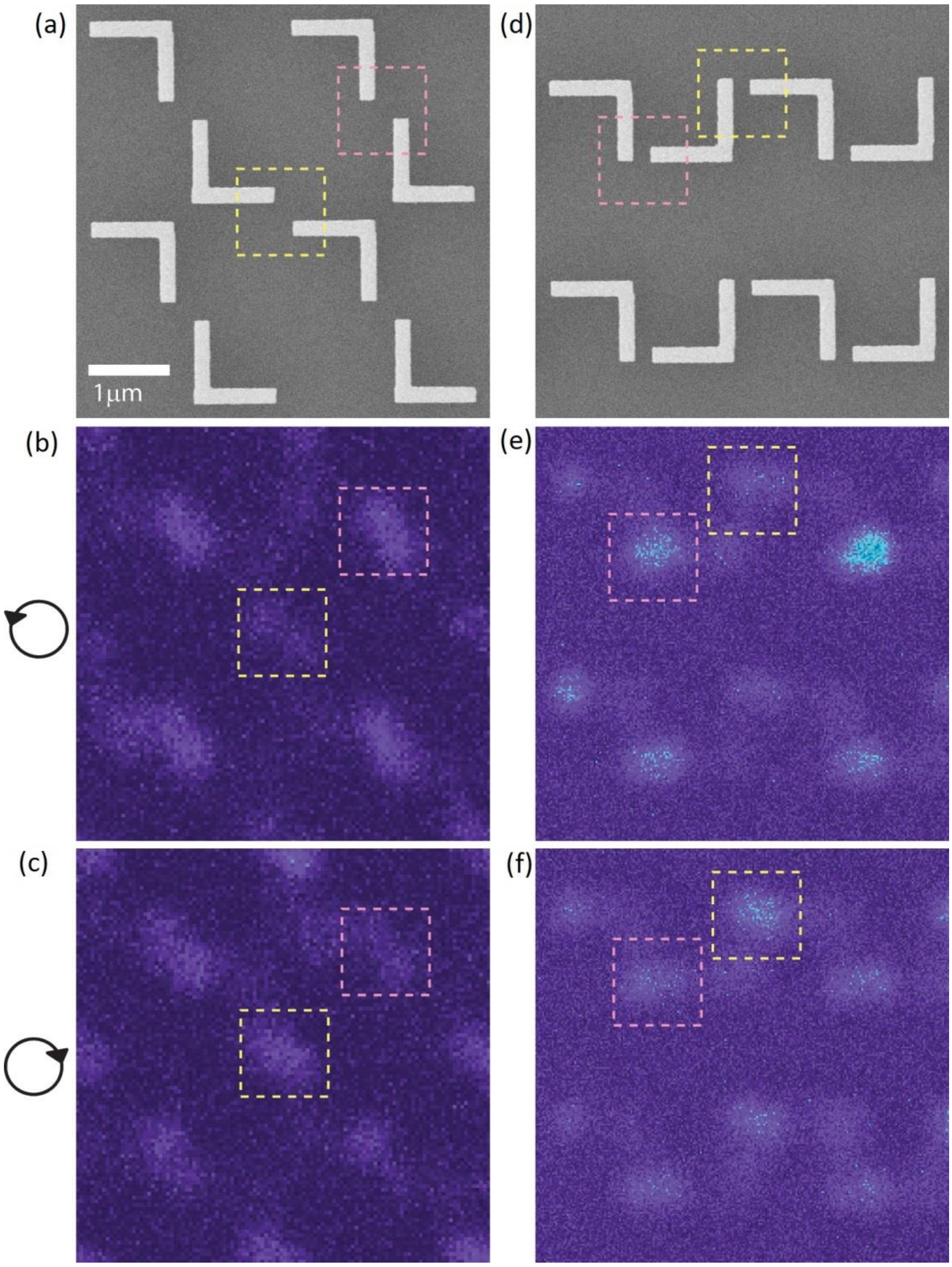

Figure S1. The chiral coupling occurs both across the diagonal and side-to-side in the unit cells. (a), SEM micrograph of the sample with diagonally coupled unit cells. The samples were 60 nm thick. (b) and (c) show SHG microscopy images for left- and right-hand circularly polarized light, respectively, illuminated at 900 nm. The data are organized in a similar manner for the sample with side-to-side coupled unit cells, in (d), (e) and (f).

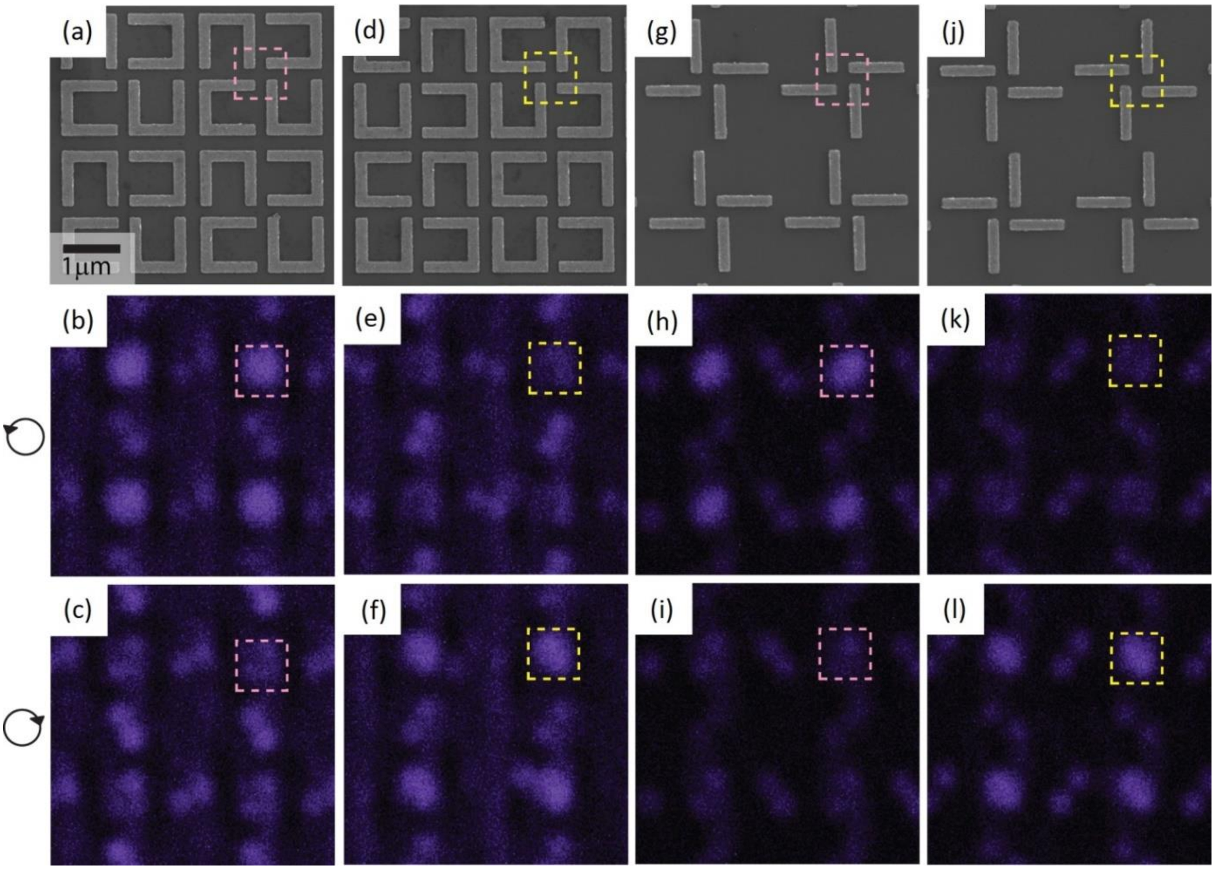

Figure S2. The chiroptical coupling at chiral centers of the nanostructured unit cells is general and can be observed for a variety of geometries. (a) SEM micrograph of the sample with U-shaped nanostructured unit cells. The gold nanostructures are 40 nm thick. (b) and (c) show SHG microscopy images for left- and right-hand circularly polarized light, respectively, at the wavelength of 800 nm. The data are organized in a similar manner for the sample with mirror-U-shaped nanostructures (in (d), (e) and (f)), for the sample with I-shaped nanostructures (in (g), (h) and (i)) and for the sample with mirror-I-shaped nanostructures. For both enantiomorphs of the U-shaped and the I-shaped nanostructures, a clear SHG-CD effect can be observed.

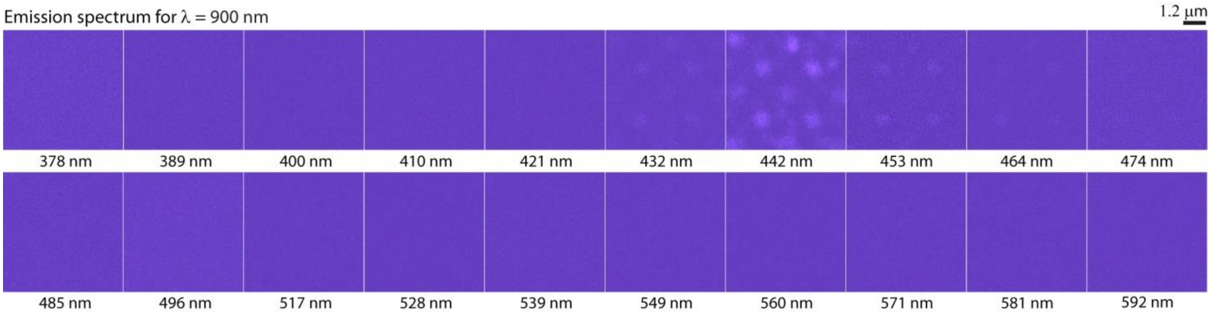

Figure S3. Two-photon luminescence does not contribute to the detected signal, which only contains the SHG. The figure shows the emission spectrum of the interspaced 卐/卍 lattices, at an illumination wavelength of 900 nm. A pattern of hotspots is visible at around 450 nm, which corresponds to the second harmonic wavelength.

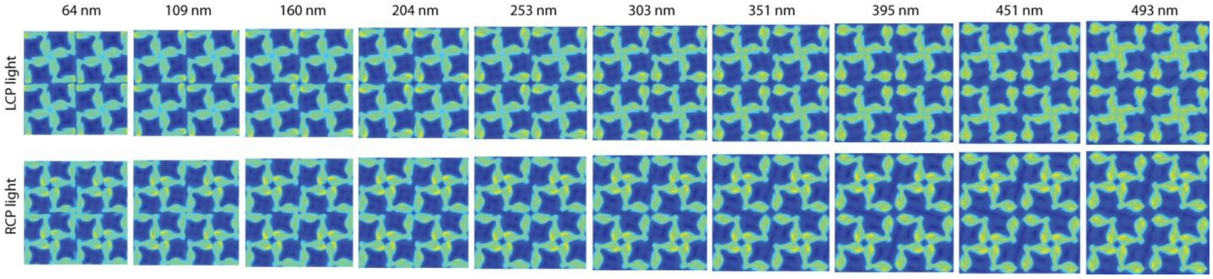

Figure S4. Electric field distribution at the fundamental frequency (900 nm), at the surface of the nanostructures. The field maps were calculated for left- and right-hand circularly polarized light and as a function of the unit cell separation distance *d*, in the samples with interspaced 卐/卍 lattices.

SHG simulations from first principle calculations:

For this, we employed a standard method used to describe the surface SHG at metal/dielectric interfaces, namely one considers that the nonlinear polarization is described by a surface nonlinear susceptibility, χ^s, via the relation:

$$\boldsymbol{P}_s(\boldsymbol{r}, 2\omega) = \chi^S : \boldsymbol{E}(\boldsymbol{r}, \omega)\boldsymbol{E}(\boldsymbol{r}, \omega)\delta(\boldsymbol{r} - \boldsymbol{r}_s), \quad \text{(S1)}$$

where $\boldsymbol{r}_s$ defines the surface. For isotropic surfaces, as in the case of noble metals, the nonlinear susceptibility tensor has only three non-vanishing components; in the case of Au their values are $\boldsymbol{\chi}^s_{\perp\perp\perp} = 1.59 \cdot 10^{-18}$ m^{2}V^{-1} and $\boldsymbol{\chi}^s_{\parallel\perp\parallel} = \boldsymbol{\chi}^s_{\parallel\parallel\perp} = 4.63 \cdot 10^{-20}$ m^{2}V^{-1}. As a commonly employed approximation, the bulk (nonlocal) contribution to the total nonlinear polarization is neglected. Under these circumstances, using equation (S1), one can determine the spatial distribution of the nonlinear polarization once the electric field at the FF is calculated. The results of these calculations, summarized in Figure 1f, clearly show that the regions where $\boldsymbol{P}_s(\boldsymbol{r}, 2\omega)$ is large can be mapped onto the regions where the density of the OAM and field chirality are enhanced as well. Since the total nonlinear dipole $\boldsymbol{p}_{L,R}$ induced in the chiral meta-molecules is given by the surface-integrated value of $\boldsymbol{P}_s(\boldsymbol{r}, 2\omega)$, one can infer that there is a direct connection between the observed SHG-CD and the density of the OAM and optical field chirality.

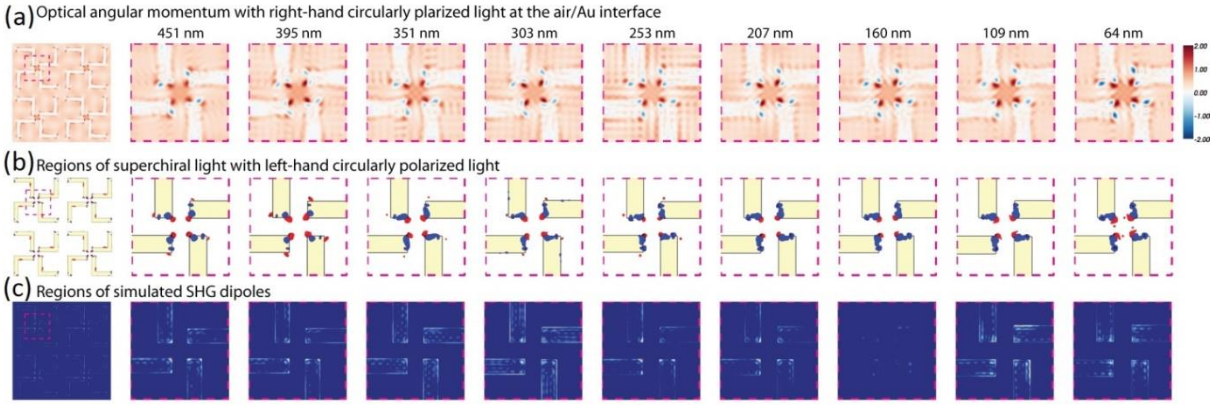

Figure S5. The chiroptical SHG signal is associated with the enhancements of optical chirality. Upon illumination with *R* light, (a) shows the distribution of the optical angular momentum at the air/Au interface. For reference, in (b), the regions of superchiral light enhancements are shown. Both the optical angular momentum and the superchiral light remain approximately constant as a function of the gap distance. (c), the numerical simulations of the SHG dipolar contribution show that the maxima of the nonlinear dipole distribution coincide with the regions of the meta-surface where a superchiral light enhancement is observed.

Reciprocity in chiral materials:

We first consider the case of circularly polarized light, interacting with a chiral object (a 3D spiral on glass substrate), see Figure S6a. Under space-reversal, a symmetry operation that is referred to as *parity* (P), *x*, *y*, *z* coordinates change to -*x*,-*y*, -*z*. As a consequence, the chiral object is transformed into its enantiomorph, the direction of light propagation reverses and the direction of polarization rotation remains the same, see Figure S6b. The whole experiment should be invariant under P and hence the resulting light-matter interaction should be identical to that in Figure S6a. For a comparison with Figure S6a, it is convenient to rotate the experimental setup by 180°. It is clear then that P reverses both the direction of circularly polarized light and the handedness of the sample, which is indeed equivalent to the initial configuration in terms of circular dichroism.

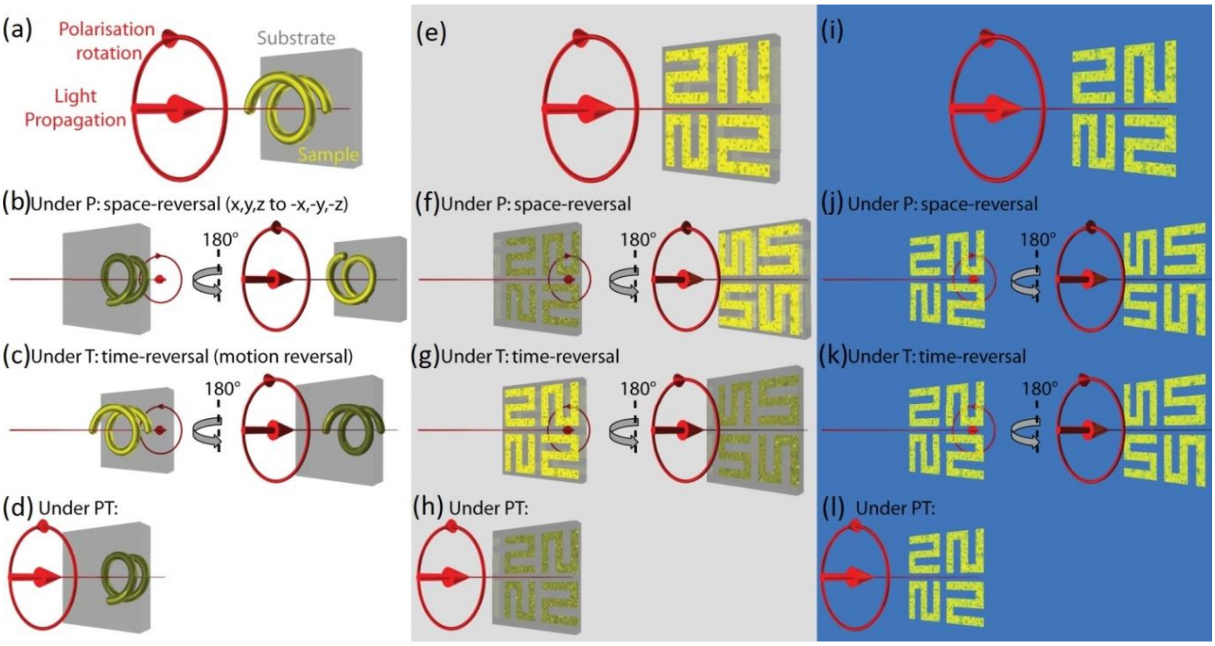

Figure S6. Reciprocity in 3D chiral structures arises from the conservation of time-reversal symmetry. (a) Circularly polarized light is incident on a chiral 3D spiral. (b), space-reversal inverts the sample and the direction of propagation but preserves the direction of polarization rotation. Upon 180° rotation, the experiment in equivalent to that in (a). (c), time-reversal reverses the direction of polarization rotation, as well as that of the light propagation, while the sample remains unchanged. Upon 180° rotation, the experiment becomes equivalent to those in (a) and (b). (d), space-time reversal reverses the direction of circular polarization and the handedness of the sample. The experiment is equivalent to that in (a), (b) and (c). The diagrams in (e), (f), (g) and (h), and those in (i), (j), (k) and (l) are organized similarly to (a), (b), (c) and (d), for the Ro symbols, with and without glass substrate, respectively.

Under time-reversal (T) symmetry, the direction of motion reverses, while the sample remains unaffected, see Figure S6c. Again a 180° rotation allows for an easier comparison with Figure S6a. It is clear then that T preserves the rotation direction of circularly polarized light. Following the rotation of the spiral, we see that T also preserves the handedness of the sample. The only difference between Figures 6a and c is the position of the glass substrate with respect to the sample. The conservation of T symmetry implies then that flipping the sample should not affect the result of the experiment, i.e. chirality is a reciprocal property. Just as P and T must be conserved in the experiment, so does their combination, see Figure S6d. A comparison between Figures S6b and d also demonstrates that reciprocity is implied.

In the case of the Ro symbol on glass substrate (Figure S6e), the situation is less intuitive than in the case of a spiral, but conservation of P (in Figure S6f), T (in Figure S6g) and PT (in Figure S6h) still implies reciprocity. In particular, a comparison between Figures

S6e and g, shows that light of the same circular polarization is incident on the samples. For T to be conserved, it is then necessary that the two sample geometries in Figures S6e and g be identical. In other words, even though we can *"see"* the Bo symbol transpiring through the glass substrate, for light, this sample still has the Ro geometry. The same is true upon considering Figures S6f and h. The glass substrate therefore seems to play an important role for reciprocity.

In Figure S6i, we consider a case where no glass substrate is present and examine the influence of P (in Figure S6j), T (in Figure S6k) and PT (in Figure S6l). Here, conservation of P seems consistent with the fact that Ro and Bo are enantiomorphs. However, under T symmetry we see non-reciprocity. Conservation of T implies that Ro and Bo should be identical, i.e. that they are not true 3D chiral objects. This observation is further confirmed upon comparing the geometries in Figures S6j and l. The glass substrate plays therefore a crucial role in the three-dimensionality of our samples.

Non-reciprocity has been reported in non-chiral photonic crystal materials.[1]

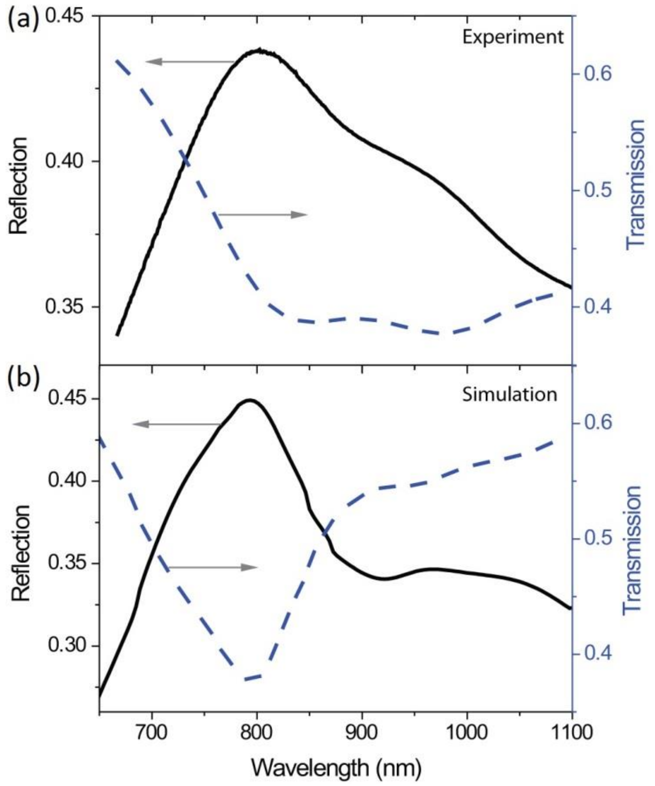

Figure S7. Linear optical spectra of the Bo nanostructures. (a), Reflection and transmission obtained experimentally. (b), The corresponding numerical simulations of reflection and transmission spectra are in good agreement with the experimental data.

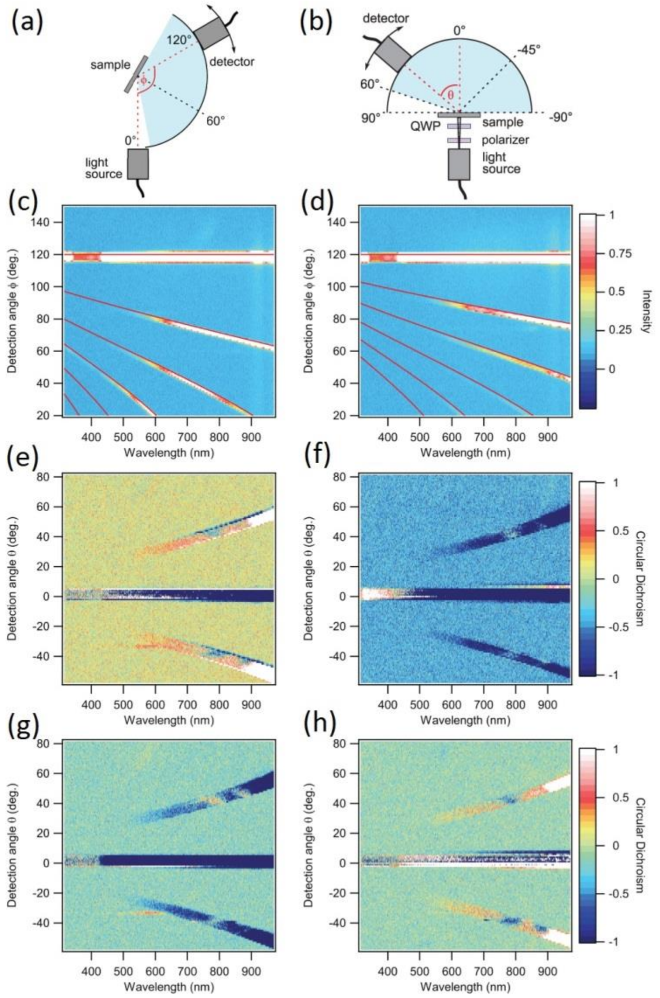

Figure S8. The first-order diffracted beams exhibit non-reciprocal chiroptical behavior. (a), the experimental geometry of the setup for characterizing the periodicity that causes diffraction. (b), the experimental geometry employed for characterizing the circular dichroism in the first-order of diffraction. (c), the diffraction pattern indicates a grating periodicity of 1200 nm for a sample oriented with the side of the Bo nanostructures along the detector plane. (d), the diffraction pattern indicates a periodicity of 1697 nm for the Bo nanostructures oriented diagonally with respect of the detector plane. (e), circular dichroism (difference between spectra acquired for *L* and *R* light) for the Bo sample, with light incident from the air/Au interface. The signal at 0° is the zero-order beam, where the CD effect is too weak and requires a CD-Spectrometer to be measured, see Figure 3b. The two first-order diffracted beams however clearly show a large CD effect. In (f), the similar data for the Ro nanostructures can be seen. The CD effect in the first-order diffracted beam reverses as expected. In (g), the results are from the Bo nanostructures with light incident from the glass/Au interface, i.e. the sample is flipped with respect to (e). With respect to (e), the first-order diffracted beams exhibit a reversed CD response, indicating a non-reciprocal behavior. This is further confirmed in (h), for the Ro nanostructures with light incident from the glass/Au interface. Again, the CD in the first-order diffracted beam is reversed with respect to that in (f), i.e. upon flipping the sample.

References

[1]. V. Yannopapas, *Phys. Rev. A* **2013**, *88*, 043837.
